# Molecular Profiling of Nasopharyngeal Carcinoma Using the AACR Project GENIE Repository

**DOI:** 10.3390/cancers17091544

**Published:** 2025-05-01

**Authors:** Beau Hsia, Asritha Sure, Roshan Dongre, Nicolas Jo, Julia Kuzniar, Gabriel Bitar, Saif A. Alshaka, Jeeho D. Kim, Bastien A. Valencia-Sanchez, Michael G. Brandel, Mariko Sato, John Ross Crawford, Michael L. Levy, Sean P. Polster, Vijay A. Patel

**Affiliations:** 1 School of Medicine, Creighton University, Phoenix, AZ 85012, USA; beauhsia@creighton.edu (B.H.);; 2 School of Medicine, Boston University, Boston, MA 02118, USA; 3 School of Engineering Medicine, Texas A&M University, Houston, TX 77030, USA; 4 Herbert Wertheim College of Medicine, Florida International University, Miami, FL 33199, USA; njo001@med.fiu.edu; 5 Rutgers New Jersey Medical School, Newark, NJ 07103, USA; 6 Department of Otolaryngology-Head and Neck Surgery, Naval Medical Center San Diego, San Diego, CA 92134, USA; 7 Department of Otolaryngology-Head and Neck Surgery, Mayo Clinic Florida, Jacksonville, FL 32224, USA; 8 Department of Neurosurgery, University of California San Diego-Rady Children’s Hospital, San Diego, CA 92123, USA; 9 Department of Pediatric Oncology, Children’s Hospital of Orange County, Orange, CA 92868, USA; 10 Department of Pediatrics and Neurology, Children’s Hospital Orange County, University of California Irvine, Orange, CA 92868, USA; john.crawford@choc.org; 11 Department of Neurosurgery, University of Chicago, Chicago, IL 60637, USA; 12 Department of Otolaryngology-Head and Neck Surgery, University of California San Diego, San Diego, CA 92093, USA; 13 Division of Pediatric Otolaryngology, Rady Children’s Hospital, San Diego, CA 92123, USA

**Keywords:** nasopharyngeal carcinomas, AACR project GENIE, somatic mutations, *KMT2D*, *TP53*, *CYLD*, p53, NF-κB, PI3K, biomarker discovery, targeted therapy, cancer genomics

## Abstract

Background: Nasopharyngeal carcinoma (NPC) is a rare head and neck cancer arising from the mucosal lining of the nasopharynx, for which systemic therapeutic options remain scarce, reflecting the limited characterization of its genomic profile. This study utilized a large patient-level genomic repository to characterize genetic alterations, identify potential therapeutic targets, and improve disease modeling in NPC. Methods: A retrospective analysis of NPC samples was conducted using the AACR Project GENIE database. Targeted sequencing data were analyzed for recurrent somatic mutations, tumor mutational burden, and chromosomal copy number variations, with significance set at *p* < 0.05. Results: Frequent mutations were identified in *KMT2D* (20%), *TP53* (16%), *CYLD* (9.6%), *NFKBIA* (6.4%), and *PIK3CA* (5.6%), implicating the p53, NF-κB, and PI3K pathways in NPC development. Notably, significantly distinct mutational profiles were observed based on both sex and race, with female patients exhibiting higher frequencies of *PIK3C2G*, *ETV6*, and *CDKN1B* mutations and non-Asian patients showing enrichment in *KDM5A*, *CCND2*, and *TP53* mutations. Conclusions: This study presents a detailed genomic profile of NPC, identifying key mutations within established cancer-associated pathways. The identification of frequently mutated pathways (p53, NF-κB, and PI3K) suggests potential targets for novel therapies. Furthermore, distinct mutational landscapes in female and Asian NPC patients offer possibilities for precision therapeutic interventions.

## Simple Summary

Nasopharyngeal carcinoma (NPC) originates from the epithelial cells of the nasopharyngeal mucosa. This study investigates the genomic landscape of NPC using a large, national, patient-level dataset from the American Association for Cancer Research (AACR) Project Genomics, Evidence, Neoplasia, Information, Exchange (GENIE). To advance the understanding of NPC biology, this research characterized the profile of somatic alterations and investigated their influence on tumor characteristics, therapeutic efficacy, and clinical prognosis. These findings suggest potential biomarkers and targets for future therapeutic strategies.

## 1. Introduction

Nasopharyngeal carcinoma (NPC) is a malignant epithelial neoplasm arising from the mucosal lining of the nasopharynx, most commonly within the fossa of Rosenmüller [1]. Histopathologically, it is classified into keratinizing, non-keratinizing, and basaloid squamous cell carcinoma [2]. Clinically, presentations are diverse and stage-dependent, ranging from asymptomatic neck masses to epistaxis, nasal obstruction, dysphagia, hoarseness, otalgia, and rhinorrhea [3]. Prognosis is variable, with an overall 5-year survival rate of approximately 63%, but this is significantly influenced by histological subtype and disease stage at diagnosis [4].

Although NPC is relatively rare globally, with an age-standardized rate (ASR) of 1.5 per 100,000 in 2020, its incidence exhibits marked geographic variation [5]. Over 80% of cases occur in Asia, with particularly high ASRs observed in China (2.4 per 100,000) compared to predominantly Caucasian populations (less than 0.2 per 100,000) [5]. Established risk factors include Epstein–Barr virus (EBV) infection, occupational exposures, tobacco and alcohol use, diet, and genetic predisposition [6]. Notably, incidence is two to three times higher in males. In low-risk populations, a bimodal age distribution is observed, with peaks in young adulthood (15–25 years) and later life (65–79 years) [7].

**Figure 1:**
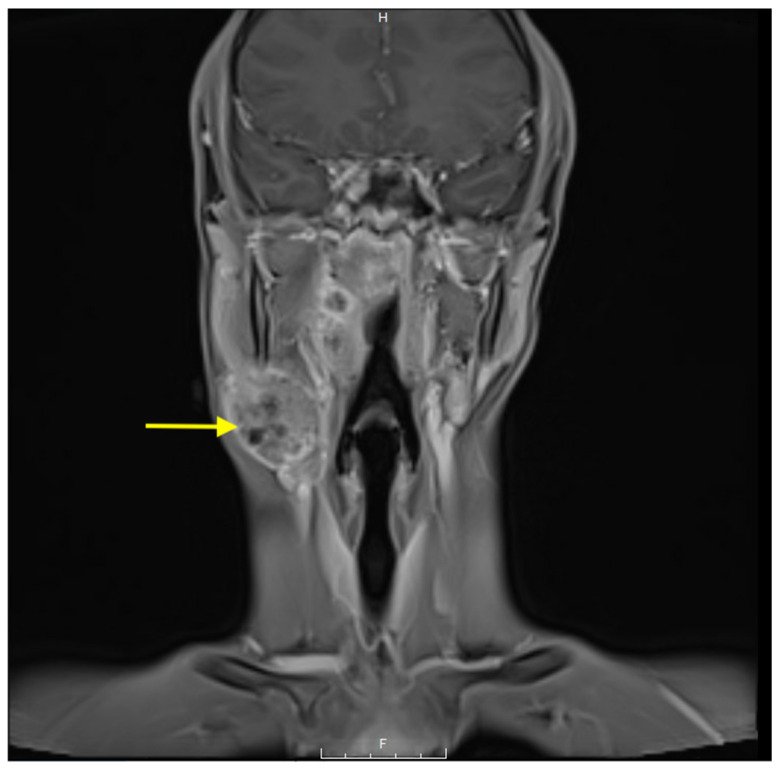
Coronal T1-weighted post-contrast MRI demonstrating a large, enhancing mass (indicated by the arrow) in the right nasopharynx, extending into the parapharyngeal space, indicative of nasopharyngeal carcinoma.

The initial workup for suspected NPC requires endoscopic examination of the nasopharynx with biopsy of any suspicious lesions and is essential for definitive diagnosis [8]. Imaging studies, such as MRI or CT scans, are crucial for evaluating the extent of the primary tumor and assessing for regional or distant metastases [9]. As seen in Figure 1, imaging can reveal the primary tumor mass and its relationship to surrounding structures.

Due to the complex anatomy of the nasopharynx, surgical resection is typically not feasible, and treatment primarily relies on platinum-based chemotherapy, radiotherapy, or concurrent chemoradiotherapy [8,9]. Emerging evidence supports the use of neoadjuvant chemotherapy followed by chemoradiotherapy, with one randomized controlled trial demonstrating a significant improvement in 5-year overall survival (87.9% vs. 78.8% with standard therapy) [10]. Further investigation into the molecular mechanisms driving NPC is crucial for developing novel therapeutic strategies, particularly targeted neoadjuvant approaches, to improve patient outcomes.

While EBV DNA has long been recognized as a valuable biomarker, recent studies have highlighted the potential of *TP53* mutations and aberrant expression of microRNAs such as miR-29 and miR-375 for molecular classification and risk stratification [11,12]. Given that the majority of NPCs present at advanced stages, and that 5-year survival rates decline from 82% in Stage I to 49% in Stage IV, the development of effective screening strategies is paramount [4,13]. Preliminary studies suggest that targeted screening programs in high-risk populations may lead to improved survival [14].

Despite these advances, a comprehensive understanding of the genetic alterations underlying NPC pathogenesis remains incomplete. Identifying additional genetic drivers of tumor progression, treatment resistance, and metastasis is essential for developing more effective diagnostic and therapeutic interventions. This study leverages a publicly available repository to provide a deeper characterization of the somatic genomic landscape of NPC, with the goal of informing the development of future therapeutics and screening methodologies.

## 2. Materials and Methods

Creighton University (Phoenix, AZ, USA) granted this study exemption from institutional review board approval due to the utilization of the de-identified and publicly accessible American Association for Cancer Research (AACR) Project Genomics Evidence Neoplasia Information Exchange (GENIE)^®^ database. Data retrieval was performed via the cBioPortal (v17.0-public) online software on 6 February 2025, encompassing clinical and genomic data archived from 2017 onwards. The AACR GENIE^®^ repository aggregates genomic sequencing data contributed by 19 international cancer centers. This dataset exhibits heterogeneity in sequencing platforms, incorporating whole-genome sequencing (WGS), whole-exome sequencing (WES), and targeted gene panels (spanning 50 to 555 genes). The distribution of sequencing platforms across the dataset is as follows: approximately 80% of samples underwent targeted panel sequencing, 15% underwent WES, and 5% underwent WGS. Variations in sequencing depth were observed across platforms: targeted panels achieved coverage exceeding 500×, WES yielded approximately 150× coverage, and WGS generated approximately 30× coverage. The sample cohort consisted of 65% tumor-only sequencing specimens and 35% matched tumor-normal pairs; the latter allowed for germline variant filtering.

While participating institutions employ institution-specific pipelines for mutation calling and annotation, adherence to the GENIE harmonization protocols is maintained according to the Genome NEXUS (e.g., utilizing GATK for variant detection and ANNOVAR for annotation, although each participating institution uses their own version of these software applications). Therapeutic response and clinical outcome data are available for a subset of cancer types within the database; however, treatment regimens were not recorded for NPC. Furthermore, it should be noted that variations in bioinformatic pipelines may exist both between and within participating institutions. Genomic sequencing is performed using either unbiased whole-genome/exome sequencing or targeted panels encompassing up to 555 genes.

The study cohort comprised patients with a pathologic diagnosis of NPC, identified from a larger group of head and neck tumor cases. Samples were designated as primary (originating from the initial tumor site) or metastatic (obtained from distant disease sites). Differences in mutation frequencies per gene between primary and metastatic tumors were evaluated using a chi-squared test based on the proportion of mutated samples in each group. The dataset comprised genomic data (e.g., somatic mutations), histological subtype, and clinical demographics (e.g., race, sex, and age). While targeted panel designs differed between institutions, key cancer-associated genes (e.g., KMT2D, TP53, PIK3CA) were covered by the majority. Non-actionable genes were generally absent from panels, and structural variants were not included in the current analysis. We assessed copy number alterations (CNAs), focusing on homozygous deletions and amplifications, and calculated the frequencies of recurrent events. Tumor mutational burden (TMB) was quantified as somatic mutations (synonymous and nonsynonymous) per megabase sequenced. Panel-derived TMB was normalized by panel size (e.g., total mutations/1.5 for a 1.5 Mb panel). These normalized values were subsequently adjusted using linear regression models developed by the AACR Project GENIE consortium to estimate whole-exome sequencing (WES)-equivalent TMB, and are available upon request to GENIE [15]. These models incorporate panel size and potentially other features to correct for panel size heterogeneity and enhance comparability across diverse sequencing platforms. Samples exhibiting missing data were excluded from the analysis. Statistical analyses were performed using R/R Studio (R Foundation for Statistical Computing, Boston, MA, USA), with statistical significance defined as *p* < 0.05. Continuous variables are presented as means ± standard deviations (SD), and categorical variables are represented as frequencies and percentages. Categorical variable associations were tested using the chi-squared test. For continuous variables, normality was assessed, and comparisons between two groups were performed using a two-sided Student’s *t*-test (normal distribution) or a Mann–Whitney U test (non-normal distribution). Adjustments for multiple comparisons were made using the Benjamini–Hochberg false discovery rate (FDR) correction.

Somatic mutations were subjected to filtering criteria that included only nonsynonymous variants (missense, nonsense, frameshift, and splice-site mutations) exhibiting a variant allele frequency (VAF) of ≥5% and sequencing coverage of ≥100×. Synonymous mutations and variants of unknown significance were excluded from the analysis. Mutation calls were derived from the GENIE harmonized mutation annotation format files. These files provide standardized variant annotation, including abbreviated gene and protein alteration, across all participating institutions.

## 3. Results

### 3.1. Patient Demographics of Nasopharyngeal Carcinoma

Due to the limited sample size of NPC within genomic cohorts, the initial demographic analysis combined primary and metastatic tumor samples. Patient demographics are detailed in Table 1. This study included 125 samples from 119 patients. Of these, 83 (69.7%) were male and 42 (35.3%) were female. Regarding ethnicity, 71 (59.7%) were non-Spanish/non-Hispanic, 9 (7.6%) were Spanish/Hispanic, and the ethnicity of 22 (18.5%) was unknown. By race, the cohort comprised 51 (42.9%) Asian, 33 (27.7%) White, 15 (12.6%) Black, and 8 (6.7%) Other. The race of 5 (4.2%) patients was unknown. The cohort included 4 (3.4%) pediatric patients and 121 (96.6%) adult patients. The average age of the adult patients was 52.2 ± 13.4 years. Of the samples, 48 (40.3%) were from primary tumors and 67 (56.3%) were from metastatic tumors. 

### 3.2. Most Common Somatic Mutations and Copy Number Alterations

**Figure 2:**
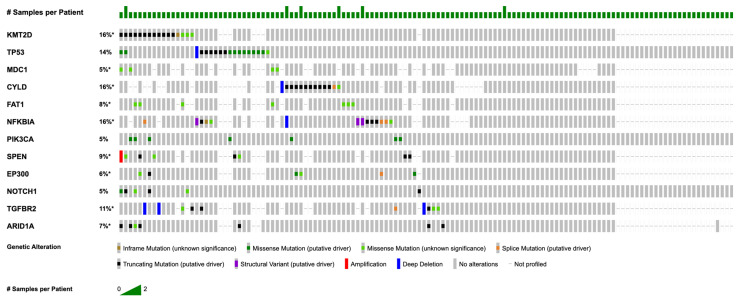
OncoPrint of recurrent mutations in NPC (for genes with *n* ≥ 5, coverage ≥ 100×, VAF ≥ 5%). Asterisk (*) denotes incomplete sample profiling.

Figure 2 summarizes somatic mutations that were most frequent in this NPC cohort. The most common mutations were identified in *KMT2D* (*n* = 25; 20.0%), *TP53* (*n* = 20; 16.0%), *CYLD* (*n* = 12; 9.6%), *FAT1* (*n* = 8; 6.4%), *NFKBIA* (*n* = 8; 6.4%), *PIK3CA* (*n* = 7; 5.6%), *SPEN* (*n* = 7; 5.6%), *MDC1* (*n* = 6; 4.8%), *EP300* (*n* = 6; 4.8%), *ROS1* (*n* = 6; 4.8%), *NOTCH1* (*n* = 6; 4.8%), *TGFBR2* (*n* = 6; 4.8%), and *AR* (*n* = 5; 4.0%). *KMT2D* mutations were the most prevalent, with mutations in *CYLD*, *FAT1*, and *NFKBIA* also observed at notable frequencies. Specific mutations for select genes are listed in Table A1. In addition to somatic mutations, we identified recurrent copy number alterations (CNAs) in 74 samples. Loss of heterozygosity (LOH) events was prevalent, particularly affecting tumor suppressor genes such as *CDKN2A* (*n* = 19; 25.7%) and *CDKN2B* (*n* = 19; 25.7%). Less prevalent were amplifications, which occurred in genes such as *FGF19*, *FGF4*, *FGF3*, and *CCND1* (*n* = 7; 10.4% for all).

### 3.3. Genetic Differences by Race and Sex

In this cohort, several mutations were uniquely observed in non-Asian patients (*p* = 0.0192), each occurring once (*n* = 1): *ASNS*, *CD40*, *CHD7*, and *ITPKB*. *TP53* mutations were also significantly enriched in non-Asian patients (*n* = 15 vs. *n* = 3; *p* = 0.0361). The differences in recurrent mutations between Asian and Non-Asian patients are highlighted in Table 2.

When stratified by sex, female patients exhibited a significant enrichment of specific mutations. Mutations in *PIK3C2G* (*n* = 5; *p* = 0.0018), *ETV6* (*n* = 4; *p* = 0.0093), and *CDKN1B* (*n* = 4; *p* = 0.0099) were exclusively found in females. Similarly, *ASNS*, *CD40*, and *CHD7* were detected only in females, each with a single occurrence (*n* = 1). *KDM5A* mutations occurred at higher frequencies in females compared to males (*n* = 5 vs. *n* = 1; *p* = 0.0137), as did mutations in *CCND2* (*n* = 5 vs. *n* = 1; *p* = 0.0149).

### 3.4. Co-Occurrence and Mutual Exclusivity of Mutations

Significant co-occurrence patterns were observed among frequently mutated genes. *KMT2D* mutations frequently co-occurred with *NOTCH1* mutations (*n* = 4/13; *p* < 0.001), *TGFBR2* (*n* = 4/11; *p* = 0.008), and *PIK3CA* (*n* = 3/13; *p* = 0.02). *CYLD* and *EP300* mutations co-occurred in two samples (*n* = 2/12; *p* = 0.056), though this was not statistically significant. *KMT2D* and *FAT1* mutations demonstrated significant co-occurrence in two cases (*n* = 2/10; *p* = 0.025). No significant mutual exclusivity patterns were identified (all comparisons showed *p* > 0.100).

### 3.5. Primary vs. Metastatic Mutations

The overall study cohort comprised 48 primary and 67 metastatic NPC cases. For the comparative genomic analysis, samples from 67 primary and 54 metastatic tumors were included. These analysis group sizes (*n* = 67 vs. *n* = 54) were of comparable size, minimizing potential bias. Among the 48 primary tumor samples, *ASNS*, *CD40*, *CHD7*, and *ITPKB* mutations were exclusively identified (*n* = 1 each; *p* = 0.0147) and were absent in the 67 metastatic samples. Conversely, *NFKB1* (*n* = 1) and *RNF213* (*n* = 1) mutations were exclusively observed in the metastatic samples (*p* = 0.04) and not detected in primary tumors. Although there were genes that were exclusively identified in primary or metastatic samples as demonstrated above, key characteristics of the mutational landscape, including tumor mutational burden (TMB) and frequencies of recurrent alterations in genes like *KMT2D*, *TP53*, and *NFKBIA*, showed substantial overlap and no significant differences between the groups.

## 4. Discussion

### 4.1. Subgroups and Mutational Landscape

This study leveraged the AACR Project GENIE repository to examine the somatic mutational landscape of nasopharyngeal carcinoma (NPC). Analysis revealed notable variations in mutation patterns across different patient subgroups. Reflecting global incidence patterns, the largest racial group in this cohort was Asian (*n* = 51), consistent with the established higher incidence of NPC among Asian/Pacific Islanders [16].

Comparative analysis identified distinct mutational profiles between Asian and non-Asian patients. *KDM5A* and *CCND2* mutations were exclusively observed in six non-Asian patients (*n* = 6). Mutations in *ASNS*, *CD40*, *CHD7*, and *ITPKB* were observed exclusively in one non-Asian patient each (*n* = 1 for each gene). Furthermore, *TP53* mutations were significantly more frequent in the non-Asian subgroup (Asian: *n* = 3, non-Asian: *n* = 15, *p* = 0.0361). While previous research has documented disparities in genetic susceptibility between Asian and non-Asian populations, including variations in HLA haplotypes and risk factors such as Epstein–Barr virus (EBV) infection [16,17], the observed enrichment of *KDM5A* and *CCND2* mutations and the exclusive presence of *ASNS*, *CD40*, *CHD7*, and *ITPKB* mutations, along with the significantly higher *TP53* mutation frequency, represent a novel finding. Notably, EBV infection rates are reportedly higher in Asian populations [18]. These findings suggest that demographic factors, including ethnicity and EBV-associated risks, contribute significantly to the distinct genetic landscapes of NPC [5,6,17].

This cohort was predominantly male (*n* = 83; 69.7%), consistent with the established 2–3-fold higher incidence of NPC in males compared to females (*n* = 42; 35.5%) [7]. Notably, female patients exhibited a higher frequency of mutations in *PIK3C2G* (5/22; 22.7%), *ETV6* (4/29; 13.8%), and *CDKN1B* (4/31; 12.9%), which were not observed in male patients. These findings highlight sex-based differences in the mutational landscape of NPC. While previous literature has acknowledged the sex disparity in NPC incidence [5,6,7], the genetic basis for these differences remains largely unexplored. This study is among the first to report these significant mutational differences between genders, underscoring the need for further research into the role of sex-specific genetic and biological factors in NPC pathogenesis. Understanding these differences may provide valuable insights into disease mechanisms and inform personalized therapeutic approaches.

### 4.2. Commonly-Mutated Genes and Known Pathways

Consistent with previous literature, our NPC cohort exhibited substantial genetic heterogeneity, with diverse mutations observed across various loci of genes implicated in multiple pathways, including cell cycle regulation, epigenetic modification, inflammation, and growth factor signaling [19]. This study identified mutations in *KMT2D* (20.0%), *TP53* (16.0%), *CYLD* (9.6%), *FAT1* (6.4%), *NFKBIA* (6.4%), *PIK3CA* (5.6%), and *SPEN* (5.6%). These findings align with prior studies identifying *KMT2D*, *TP53*, *CYLD*, *NFKBIA*, and *PIK3CA*—and their respective roles in the p53, NF-κB, and PI3K pathways—as frequently altered in NPC, further supporting their critical roles as potential driver mutations [20,21].

The efficacy of standard chemotherapies, such as platinum-based agents and 5-FU, can be, at least partially, attributed to their interaction with these commonly mutated pathways [22,23]. These agents induce significant DNA damage, which robustly activates the p53 pathway (when functional) to trigger cell death, and even in cases of p53 mutation, the overwhelming damage can surpass the cell’s repair capacity and bypass the pro-survival effects from aberrant NF-κB and PI3k signaling [24].

Interestingly, this analysis did not reveal distinct mutation types or hotspots specific to NPC, consistent with reports suggesting that NPC is a unique cancer type with few clearly defined driver events [25]. This observation underscores the role of EBV infection in NPC tumorigenesis; however, the high prevalence of EBV infection coupled with the relatively low incidence of NPC suggests a contributing role for genetic susceptibility [26]. The heterogeneous mutation patterns observed in these studies [24,25] emphasize the absence of a single unifying driver mutation, suggesting a polygenic contribution to NPC development. While this genetic diversity complicates therapeutic targeting, it also presents a broad spectrum of potential molecular vulnerabilities for exploration.

### 4.3. p53 Pathway

The tumor suppressor gene *TP53* has been extensively studied in NPC [19,21,27,28,29,30,31,32,33,34,35,36]. Consistent with these reports, we observed *TP53* mutations in 16% of our samples, representing the pathway with the greatest mutational burden in this cohort. *TP53* mutations typically occur early in tumorigenesis, disrupting G1/S checkpoint surveillance and facilitating the accumulation of subsequent oncogenic alterations [25].

Aberrant epigenetic modification is also recognized as a significant factor in NPC development [19,27,28,29,33,37]. In line with this, *KMT2D*, a key regulator of chromatin remodeling and implicated in *p53* activation, was the most frequently mutated gene in our samples (20%). Mutations affecting *KMT2D*, or its related isoform *KMT2C*, have been consistently reported in other NPC studies [19,27,33]. Notably, the frequency of *EP300* mutations, a histone acetyltransferase, was similar in our cohort (5%) compared to previous reports (4.8%) [27].

While *TP53* mutations are thought to act as an early driver of tumor initiation, mutations in chromatin organization pathways, such as *KMT2D*, are suggested to emerge later and contribute to phenotypic evolution and adaptability of NPC [25]. These findings collectively suggest that *TP53* mutations not only initiate tumorigenesis but also influence the mutational landscape, paving the way for the development of later-stage alterations crucial for tumor progression.

Targeting *TP53* pathway alterations also holds promise. Therapies in development include Gendicine, a gene therapy delivering functional *TP53*, and APR-246, a small molecule aimed at restoring p53 function in p53-deficient cancers [19,38,39,40]. Additionally, MDM2 inhibitors are being explored to inhibit p53 ubiquitination, potentially stabilizing its tumor-suppressor activity [19,38,41]. Ongoing clinical trials [NCT03745716, NCT04214860, NCT03634228, NCT03107780, NCT04485260] are investigating the efficacy of these therapies in TP53-mutated malignancies [38,39,40].

### 4.4. NF-κB Pathway

The NF-κB pathway, implicated in NPC and other diseases (e.g., inflammatory bowel disease, rheumatoid arthritis, lupus) [42], has a debated role in NPC tumorigenesis, with some studies suggesting it as a primary driver and others as part of a broader mutational landscape. Some studies [28,30] describe NPC as largely defined by NF-κB activation (40–90% of tumors), whereas other studies [18,32,33] report greater mutational diversity and lower NF-κB mutation prevalence (7–12%). The present study supports the significance of NF-κB, with *CYLD* and *NFKBIA* mutations identified in 9.6% and 6.4% of tumors, respectively, consistent with prior studies [19,27,28,29,30,31,32,33,35,43]. *NFKB1* (*n* = 1) was exclusively observed in the metastatic samples (*p* = 0.04). Although no mutations in other NF-κB pathway components (e.g., *TRAF2*, *TRAF3*, *NLRC5*) were detected, mutated NF-κB proteins remain promising druggable targets, particularly for metastatic NPC [19,28,29,31,33,43].

### 4.5. PI3K Pathway

The PI3K pathway, involved in cellular responses to various growth factors (e.g., IGF), is another well-established hotspot of mutation in NPC [19,21,27,28,29,30,32,33,34,37,44,45]. *PIK3CA* mutations were identified in 5.6% of analyzed samples. Similarly, another study [34] found 7% *PIK3CA* mutations and reported that approximately 15% of tumors harbored at least one mutated protein in the PI3K pathway, including *PIK3CA*, *PCG1*, *EGFR*, *PTEN*, and *PRKCZ*. Although representing an incomplete portion of total NPC cases, the PI3K pathway is a potentially druggable target warranting further exploration in susceptible NPC. *PIK3CA*-specific inhibitors, such as BYL719, are already under investigation for treating other *PIK3CA*-mutated tumors and *PIK3CA*-related overgrowth syndromes (e.g., CLOVES) [46,47]. Additionally, the efficacy of everolimus for solid *PIK3CA*-mutated tumors is under investigation [34].

### 4.6. Co-Occurrence Patterns and Functional Implications

The observed co-occurrence of *KMT2D* mutations with *PIK3CA* and *FAT1* suggests potential cooperative roles in NPC progression. This aligns with broader findings in other cancers, where *PIK3CA* mutations are commonly associated with pathways promoting cell survival and proliferation [48,49]. This contrasts with findings in some studies that suggest an association between *TP53/HERC1* and *KMT2D* mutations [25]. In NPC, combination therapies targeting *PIK3CA* and its co-mutated partners might be an avenue for improved outcomes, as mutations in *PIK3CA*, particularly hotspot variants like H1047R, are often linked to therapy resistance and poor outcomes in other cancers [50].

While our data did not reveal significant mutual exclusivity patterns, 98% of cases exhibited mutations in only one of the three most frequently mutated genes (*KMT2D*, *TP53*, and *CYLD*). This observation contrasts with findings in some studies that have demonstrated mutual exclusivity between LMP1 expression and NF-κB mutations or have not found any mutually exclusive driver pairs [25,29]. These discrepancies underscore the complexity of NPC’s genetic landscape and suggest that the interplay of mutational patterns may be context-dependent, influenced by cohort characteristics, environmental factors, and tumor heterogeneity, warranting further exploration in diverse populations.

### 4.7. Limitations

This study has several limitations. First, the AACR Project GENIE database lacks transcriptomic data, which is particularly relevant in NPC given the reported role of gene overexpression, even in the absence of mutations, especially for genes involved in epigenetic modification [27]. The absence of transcriptomic data precluded the correlation of mutational status with downstream pathway activity or gene expression levels. Similarly, the potential of tumor suppressor miRNAs as diagnostic and prognostic tools in NPC could not be explored due to the absence of miRNA data [37]. Second, treatment information is not included within this repository, which would have allowed the analysis of treatment response with mutational status and histologic subtype. GENIE’s lack of treatment data also precludes analysis of therapy-related genomic changes that may confound comparisons between primary and metastatic tumors. Third, because the database aggregates data from multiple centers using diverse sequencing platforms, potential inconsistencies in sequencing protocols could introduce bias in estimated mutation rates. Fourth, this study could not examine the crucial influence of DNA methylation on epigenetic control within NPC, nor its potential implications for tumor behavior and treatment response, as methylation data were not generated. Incorporating such data could offer deeper biological understanding. Fifth, the statistical robustness needed to definitively link specific genetic alterations with patient outcomes or distinct disease features in NPC was constrained by the modest cohort size. Therefore, validating mutations as independent predictors of prognosis necessitates subsequent investigations involving larger patient groups with standardized annotations. Sixth, distinguishing functionally significant driver mutations from passenger alterations accumulated during tumor evolution was impeded by the study design, which lacked serially collected samples (e.g., matched primary/metastatic tissues) from the same individuals over time. Seventh, the potential confounding effect of including a minor subset of related samples within the GENIE cohort (such as multiple tumors from one patient) is acknowledged, although prior evaluations suggest this factor likely exerts minimal influence on the principal results reported. Eighth, an inability to associate the detected mutation patterns with patient survival endpoints (including overall or disease-free survival) stems from the lack of such clinical outcome information within the specific GENIE data utilized for this NPC analysis. Ninth, the database aggregates all NPC histological subtypes (keratinizing, non-keratinizing, and basaloid squamous cell carcinoma) into a single group, preventing analysis of potential subtype-specific mutational profiles and their respective clinical implications. Ascertaining the impact of repeatedly observed mutations on patient prognosis in NPC necessitates future research that pairs genomic characterization with clinical follow-up data collected over time. An additional constraint was the inability to investigate potential links between specific genetic alterations and the protein expression patterns of diverse tumor-cell or immune-related markers via immunohistochemistry. Acknowledging these constraints, the current analysis still yields important information concerning the profile of genomic alterations typically found in NPC, highlighting the importance of several known pathways such as the p53, NF-κB, and PIK3 pathways, and identifying potential new targets for therapeutic intervention.

## 5. Conclusions

This study provides a comprehensive analysis of the NPC mutational landscape using the AACR Project GENIE database, revealing frequent alterations in *KMT2D*, *TP53*, *CYLD*, *NFKBIA*, and *PIK3CA*, key components of the p53, NF-κB, and PI3K pathways. Notably, distinct mutational patterns based on both sex and race were identified, highlighting the potential for personalized therapeutic strategies tailored to specific patient populations. These findings advance understanding of NPC biology and may inform the development of more effective preclinical models, diagnostic testing, and targeted therapies.

## Figures and Tables

**Table 1:** Nasopharyngeal carcinoma patient demographics.

Demographics	Category	*n* (%)
Sex	Male	83 (69.7)
	Female	42 (35.5)
Age category	Adult	121 (96.6)
	Pediatric	4 (3.4)
Ethnicity	Non-Hispanic	71 (59.7)
	Unknown/Not Collected	22 (18.5)
	Hispanic	9 (7.6)
Race	Asian	51 (42.9)
	White	33 (27.7)
	Black	15 (12.6)
	Other	8 (6.7)
	Unknown	5 (4.2)
Sample Type	Primary	48 (40.3)
	Metastasis	67 (56.3)
	Not Collected	6 (5.0)

**Table 2:** Race and associated mutations.

Gene (Chi-Squared)	Asian, *n* (%)	Non-Asian, *n* (%)	*p* Value
*TP53*	3 (5.9)	15 (12.0)	*p* = 0.0361
*CCND2*	0 (0.0)	6 (4.8)	*p* = 0.0272
*KDM5A*	0 (0.0)	6 (4.8)	*p* = 0.0259
*ITPKB*	0 (0.0)	1 (0.8)	*p* = 0.0192
*CD40*	0 (0.0)	1 (0.8)	*p* = 0.0192
*CHD7*	0 (0.0)	1 (0.8)	*p* = 0.0192
*ASNS*	0 (0.0)	1 (0.8)	*p* = 0.0192
	**Male, *n* (%)**	**Female, *n* (%)**	
*KDM5A*	1 (0.8)	5 (4.0)	*p* = 0.0137
*CCND2*	1 (0.8)	5 (4.0)	*p* = 0.0149
*PIK3C2G*	0 (0.0)	5 (4.0)	*p* = 0.0018
*ETV6*	0 (0.0)	4 (3.2)	*p* = 0.0093
*CDKN1B*	0 (0.0)	4 (3.2)	*p* = 0.0099
*ASNS*	0 (0.0)	1 (0.8)	*p* = 0.0147
*CD40*	0 (0.0)	1 (0.8)	*p* = 0.0147
*CHD7*	0 (0.0)	1 (0.8)	*p* = 0.0147

## Data Availability

The data presented in this study are available from the AACR GENIE Database at https://genie.cbioportal.org/ (accessed on 6 February 2025).

## Abbreviations

American Association for Cancer Research (AACR), asparagine synthetase (*ASNS*), cyclin D2 (*CCND2*), CD40 molecule (CD40), cyclin-dependent kinase inhibitor 1B (*CDKN1B*), chromodomain helicase DNA binding protein 7 (*CHD7*), copy number alterations (CNAs), CYLD lysine 63 deubiquitinase (*CYLD*), deoxyribonucleic acid (DNA), Epstein–Barr Virus (EBV), E1A binding protein P300 (EP300), ETS variant transcription factor 6 (*ETV6*), FAT atypical cadherin 1 (*FAT1*), Genomics, Evidence, Neoplasia, Information, Exchange (GENIE), inositol-trisphosphate 3-kinase B (*ITPKB*), lysine demethylase 5A (*KDM5A*), lysine methyltransferase 2D (*KMT2D*), microRNA (miRNA), nuclear factor kappa-light-chain-enhancer of activated B cells (NF-κB), nuclear factor kappa B subunit 1 (*NFKB1*), NFKB inhibitor alpha (NFKBIA), notch receptor 1 (*NOTCH1*), nasopharyngeal carcinoma (NPC), tumor protein p53 (p53), phosphoinositide 3-kinase (*PI3K*), phosphatidylinositol-4-phosphate 3-kinase catalytic subunit type 2 gamma (*PIK3C2G*), phosphatidylinositol-4,5-bisphosphate 3-kinase catalytic subunit alpha (*PIK3CA*), standard deviation (SD), spen family transcriptional repressor (*SPEN*), transforming growth factor beta receptor 2 (*TGFBR2*), tumor mutational burden (TMB), tumor protein P53 (*TP53*), and variant allele frequency (VAF).

## Appendix A

**Table A1:** Specific mutations for the select genes in NPC. Asterisk (*) indicates a premature stop codon.

Gene	Protein Change	Mutation Type
*KMT2D*	M4159Cfs*2	FS del
	S2431Lfs*53	FS del
	A2119Lfs*25	FS del
	L3542Vfs*13	FS del
	C5226Afs*16	FS del
	P648Tfs*2	FS ins
	L2331Pfs*46	FS ins
	Q3863del	IF del
	E2393K	Missense
	D32N	Missense
	R4420W	Missense
	I3065M	Missense
	K2288N	Missense
	L894F	Missense
	Q4249H	Missense
	D1724Y	Missense
	A2925V	Missense
	R1615 *	Nonsense
	Q3441 *	Nonsense
	W4987 *	Nonsense
	Q2416 *	Nonsense
	E4662 *	Nonsense
	Q4609 *	Nonsense
	S3239 *	Nonsense
	X4882_splice	Splice
*TP53*	K382Nfs*40	FS del
	L257Gfs*6	FS del
	P71Rfs*87	FS ins
	Y220C	Missense
	Y220C	Missense
	R248Q	Missense
	R273C	Missense
	Y163C	Missense
	E285K	Missense
	P278S	Missense
	V157F	Missense
	V157F	Missense
	E271K	Missense
	C238G	Missense
	G245A	Missense
	E349K	Missense
	R213 *	Nonsense
	Q165 *	Nonsense
	R196 *	Nonsense
	X307_splice	Splice
*CYLD*	E585Rfs*2	FS del
	P910Qfs*3	FS del
	Q443Pfs*4	FS ins
	I161Efs*43	FS ins
	D681N	Missense
	Q898 *	Nonsense
	Q554 *	Nonsense
	S371 *	Nonsense
	S371 *	Nonsense
	E626 *	Nonsense
	Q823 *	Nonsense
	X896_splice	Splice
*NFKBIA*	R264Pfs*21	FS ins
	D100Rfs*29	FS ins
	K238Efs*5	FS ins
	E14Gfs*72	FS ins
	NFKBIA intragenic	Fusion
	NFKBIA intragenic	Fusion
	NFKBIA intragenic	Fusion
	A158_N182del	IF del
	G161E	Missense
	X113_splice	Splice
	X154_splice	Splice
